# *Staphylococcus aureus* α-Toxin-Dependent Induction of Host Cell Death by Membrane-Derived Vesicles

**DOI:** 10.1371/journal.pone.0054661

**Published:** 2013-01-31

**Authors:** Bernard Thay, Sun Nyunt Wai, Jan Oscarsson

**Affiliations:** 1 Oral Microbiology, Department of Odontology, Umeå University, Umeå, Sweden; 2 Department of Molecular Biology and the Umeå Centre for Microbial Research (UCMR), Umeå University, Umeå, Sweden; National Institutes of Health, United States of America

## Abstract

*Staphylococcus aureus* causes a wide spectrum of infections in humans, ranging from superficial cutaneous infections, infections in the circum-oral region, to life-threatening bacteremia. It was recently demonstrated that Gram-positive organisms such as *S. aureus* liberate membrane-derived vesicles (MVs), which analogously to outer membrane vesicles (OMVs) of Gram-negative bacteria can play a role in delivering virulence factors to host cells. In the present study we have shown that cholesterol-dependent fusion of *S. aureus* MVs with the plasma membrane represents a route for delivery of a key virulence factor, α-toxin (α-hemolysin; Hla) to human cells. Most *S. aureus* strains produce this 33-kDa pore-forming protein, which can lyse a wide range of human cells, and induce apoptosis in T-lymphocytes. Our results revealed a tight association of biologically active α-toxin with membrane-derived vesicles isolated from *S. aureus* strain 8325-4. Concomitantly, α-toxin contributed to HeLa cell cytotoxicity of MVs, and was the main vesicle-associated protein responsible for erythrocyte lysis. In contrast, MVs obtained from an isogenic *hla* mutant were significantly attenuated with regards to both causing lysis of erythrocytes and death of HeLa cells. This is to our knowledge the first recognition of an *S. aureus* MV-associated factor contributing to host cell cytotoxicity.

## Introduction

Many Gram-negative bacterial species can extend their pathogenicity by releasing outer membrane vesicles (OMVs), which can expose host cells to relatively high concentrations of toxins and additional virulence factors without the requirement of a close contact between the bacterial and target human cells [1]–[7]. OMVs may therefore have a pivotal role in effecting a toxic response in the host towards the bacterial pathogens. In recent years it has become evident that also Gram-positive organisms, i.e. *Staphylococcus aureus*, *Mycobacterium ulcerans*, and *Bacillus spp*. liberate membrane-derived vesicles (MVs) [8]–[10], suggesting that vesicle production may be a property of most bacterial species.

A wide spectrum of infections in humans is caused by *S. aureus*, ranging from superficial cutaneous infections, infections in the circum-oral region, to deep systemic infections, such as osteomyelitis, endocarditis, and bacteremia. Combined with its distinct ability to acquire resistance to multiple antibiotics, *S. aureus* is one of the major dangers to human health [11], [12]. The extensive range of diseases caused by this organism is likely a consequence of its many virulence factors, which include surface proteins, and disseminated enzymes, and toxins. Particularly important is α-toxin (α-hemolysin; Hla), which is produced by most *S. aureus* strains. This is a 33-kDa protein that forms heptameric pores in target cell membranes [13], [14]. Secreted α-toxin has cytolytic and/or cytotoxic activity against many mammalian cell types [15]–[17]. A series of events can be provoked by α-toxin depending on the target cell species and the dosage of the toxin. High concentrations of the toxin mainly produce necrosis of the target cells, whereas sublytic concentrations predominantly induce apoptosis [18]–[20], and/or an inflammatory response in the target cells [21]. A disintegrin and metalloprotease 10 (ADAM10) has been identified as the likely proteinaceous host cell receptor for α-toxin [22].

Interestingly, proteomics analyses of *S. aureus* MV preparations identified a plethora of proteins and enzymes, including α-toxin and the IgG-binding protein A (SPa) [8], [23]. Consistent with these observations, MVs were found to induce atopic dermatitis-like skin inflammation *ex vivo* [24], and to induce cytotoxicity on human laryngeal carcinoma (Hep-2) cells [23]. The MV-induced toxic effects appeared to be associated with translocation of *S. aureus* effector proteins to the host cells. Analogously to OMV-mediated transport of virulence-related proteins, plasma membrane cholesterol was required for delivery of *S. aureus* virulence factors (i.e. protein A) to MV-treated Hep-2 cells [23]. Hence, *S. aureus* membrane-derived vesicles may represent an important vehicle to expose host cells to multiple effector proteins. To further understand the mode of interaction of *S. aureus* MVs with human cells, and to make an attempt to reveal the identity of MV-associated factor(s) causing cytotoxicity, we have assessed whether α-toxin may be released via MVs.

## Materials and Methods

### Bacterial strains and growth conditions

*S. aureus* prototype strain 8325-4 (*rsbU*) [25], and its derivative DU1090 (*hla*::ermB; [Em^r^]) [26] were mainly used in the present study. *S. aureus* WA764 belongs to the strain collections of Dr. Staffan Arvidson, Karolinska Institutet, Stockholm, who kindly donated it to us. This strain is a *spa* mutant (*spa*::ermB; [Em^r^]) construct of RN4220, a restriction-deficient derivative of 8325-4 [27]. The strains were grown aerobically at 37°C on blood agar plates (5% defibrinated horse blood, 5 mg hemin/l, 10 mg Vitamin K/l, Columbia agar base), or in Luria-Bertani (LB) broth.

### Cell lines, culturing and treatment conditions

HeLa cells (ATCC number CCL-2) were cultured in Advanced Minimum Essential Medium (MEM) (Invitrogen) supplemented with 2 mM glutamine, 10% fetal calf serum, and 50 µg/ml gentamycin. The cells were cultivated at 37°C in a 5% CO_2_ atmosphere in air. Prior to experimental treatment, cells were washed with PBS and treated with trypsin (250 µg/ml in PBS) for 5 min at 37°C. Trypsin-treated cells were collected by centrifugation and then resuspended in MEM medium containing gentamicin (final concentration 50 µg/ml).

### Isolation of membrane-derived vesicles

MVs were isolated from culture supernatants essentially following procedures described earlier [7], [23]. Briefly, *S. aureus* strains were grown in 500 ml LB-medium and cultivated to an optical density at 600 nm (OD_600_) of 2.0. After removal of the bacterial cells by centrifugation (12,000× *g*; 30 min, 4°C) in a JLA 10-500 rotor (Beckman Instruments Inc.), supernatants were filtered (0.22 μm; Millipore) and then centrifuged at 85,000× *g* (2 h, 4°C) in a 70 Ti rotor (Beckman Instruments Inc.) to collect MVs. Pellets were washed twice with PBS (85,000× *g*; 2 h, 4°C), then suspended in PBS and used as the MV preparation. The amounts of vesicles were estimated by quantifying MV preparations for protein content using a Picodrop™ (Picodrop Ltd.). Preparations of membrane-derived vesicles were checked for absence of bacterial contamination by cultivating small aliquots on blood agar. Fractionation of MV preparations was performed by density gradient centrifugation essentially as described earlier [28], [29]. In this procedure vesicles migrate to positions in the gradient that are equal to their density, and only proteins integral, internal, or tightly associated with membrane lipids will move significantly through the gradient [28]. For this, MV pellets were resuspended in 50 mM HEPES (pH 6.8), and then adjusted to 45% Optiprep (Sigma-Aldrich) in a final volume of 150 µl. The sample was transferred to the bottom of a 4-ml ultracentrifuge tube, and then different Optiprep/HEPES layers were sequentially added as follows: 900 µl 35%, 900 µl 30%, 660 µl 25%, 660 µl 20%, 400 µl 15%, and 500 µl 10%. Gradients were centrifuged at 180,000× *g* (3 h, 4°C) in a SW 60 Ti rotor (Beckman Instruments Inc.), and fractions of equal volumes (200 µl) were removed sequentially from the top.

### Dissociation assay

A dissociation assay was carried out essentially as described previously [2], [29]. In brief, MV preparations in PBS (approximately 10 µg/ml total protein) were treated with PBS, 0.8 M and 8 M urea, and 1% SDS, respectively (60 min on ice). Samples were then centrifuged at 120,000× *g* (2 h, 4°C) in a SW 60 Ti Rotor (Beckman Instruments Inc.). The resulting supernatants were acetone precipitated and resolubilized in an equal volume of PBS as the pellets. Both pellets and supernatants were subsequently analyzed by Western immunoblotting.

### SDS-PAGE and Western immunoblotting

The procedures used for SDS-PAGE and Western immunoblot analysis have been described previously [30], [31]. For immunoblot detection, we used a polyclonal antiserum raised in rabbit against *S. aureus* α-toxin (Sigma-Aldrich; final dilution 1∶5,000). Protein A was fortuitously detected due to its IgG-binding properties. In accordance with earlier studies both full-length and degraded forms of protein A were detected [23]. CodY was detected using a polyclonal antiserum raised in rabbit against the *Bacillus subtilis* CodY protein (1∶2,000) [32]. Horseradish peroxidase (HRP)-conjugated anti-rabbit secondary antibodies (Jackson ImmunoResearch Laboratories Inc.) were used at a final dilution of 1∶10,000. Immunoreactive bands were visualized using SuperSignal® (Pierce) and the ChemiDoc™ XRS+ System (Bio-Rad). Densitometric analyses of band density were carried out using ImageJ software.

### Electron microscopy

For analysis by electron microscopy, MVs were resuspended in 10 mM Tris-HCl buffer (pH 7.4) containing 10 mM MgCl_2_, and allowed to adhere to Formvar-coated grids for 5 min at RT. The grids were incubated with rabbit polyclonal antibodies specific for α-toxin or CodY (final dilution 1∶500) for 10 min at RT. After the incubation, the grids were thoroughly rinsed with buffer and incubated with 10-nm gold-coupled goat anti-rabbit secondary antibodies (Biocell GAR10; 1∶20) for 10 min at RT. The grids were then rinsed in distilled water and negatively stained with 1% sodium silicotungstate (TAAB Laboratories Equipment Ltd.). Grids were examined using a Jeol 1230 transmission electron microscope, and digital images were captured using a Gatan MSC 600CW multiscan camera.

### Atomic Force Microscopy

For atomic force microscopy (AFM), bacterial cells were suspended in ultrapure water (Millipore) and 10 ml bacterial suspension was then placed on a freshly cleaved mica surface. The samples were incubated for 5 min at room temperature and blotted dry before being placed into a desiccator for at least 2 h. Imaging was performed using a Nanoscope V Atomic Force Microscope (Bruker AXS) using Tapping Mode with standard silicon cantilevers. Final images were plane fitted in both x and y axes and are presented in amplitude mode.

### Hemolysis assay

Quantitative hemolytic assays were performed essentially as described earlier [33]. In brief, 50 µl of a rabbit erythrocyte suspension in PBS was mixed with an equal volume of MVs or PBS in 96-well microtiter plates. Where applicable, vesicles were disrupted by 2 min sonication (30-second pulses; 30% amplitude) prior to being used in hemolysis assays. After 60 min incubation at 37°C, 100 µl of ice-cold PBS was added to the wells prior to centrifugation (400× *g*; 15 min, 4°C). The hemolytic activity was determined by the release of hemoglobin, measured spectrophotometrically at 540 nm.

### HeLa cell cytotoxicity assays

To monitor MV-associated cytotoxic effects, 5×10^5^ HeLa cells were treated with different concentrations of *S. aureus* MVs for 24 h (final MV protein concentrations 10–50 µg/ml). To estimate the proportion of viable cells after the treatment with membrane-derived vesicles, a neutral red uptake assay was carried out as described previously [34]. This procedure is based on the ability of viable cells to incorporate and bind the supravital dye neutral red in the lysosomes. Alternatively, to quantitate the proportion of dead cells, the treated HeLa cells were stained with fluorescein isothiocyanate (FITC)-conjugated Annexin V and propidium iodide (PI) (BD Biosciences). The samples were then immediately analyzed by flow cytometry using CellQuest Pro software (BD Biosciences), acquiring 5×10^4^ cells for data analysis. To confirm that α-toxin was delivered to HeLa cells, cell aliquots of 1 ml (1×10^6^ cells per ml) were incubated with different amounts of *S. aureus* MV preparations for 4 h and 24 h (final MV protein concentrations 10–50 µg/ml). Treated HeLa cell samples were thoroughly washed three times with PBS to remove loosely attached proteins. The cells were then lysed in 1× Laemmli sample buffer containing 200 mM Dithiothreitol (DTT) (Sigma-Aldrich) and assessed by Western immunoblotting.

### Membrane fusion assay

To assess membrane fusion using *S. aureus* MVs and HeLa cells, we essentially followed procedures described earlier [29], [35]. In brief, MVs were labeled with rhodamine isothiocyanate B-R18 (Molecular probes) at a saturated concentration (1 mg/ml) for 1 h at room temperature. Fluorescence of this probe is quenched at high concentrations in bilayer membranes, and dequenched when the probe is diluted due to membrane fusion [35], [36]. Unlabeled probe was removed by centrifugation at 100,000× *g* (60 min, 4°C). After a washing step (PBS), B-R18-labeled MVs were resuspended in 1 ml PBS (0.2 M NaCl). Subsequently, the host cell plasma membrane was labeled with FITC-conjugated cholera toxin B subunit (CtxB) (Sigma-Aldrich) at a concentration of 8 µg/ml for 1 h prior to the incubation with MVs. CtxB binds to lipid raft-enriched G_M1_ ganglioside in the plasma membrane and is widely exploited as a marker to visualize lipid rafts [37]. B-R18-labeled MVs were then applied to the apical side of HeLa cells so that the MVs were diluted 1∶4 relative to the volume of MEM medium in the wells. Cells were incubated with MVs for 30 min at 37°C. When applicable, the cholesterol-sequestering agent Filipin III [38] was added at a final concentration of 10 µg/ml 30 min prior to the addition of MVs. After the incubation with MVs, cell samples were analyzed by confocal microscopy as described below.

### Confocal microscopy

For analysis by confocal microscopy, coverslips were mounted with Mowiol (Scharlau Chemie S.A.) containing antifade (P-phenylene diamine). Confocal microscopy was carried out using a NIKON D-Eclipse C1 Confocal Laser with a NIKON Eclipse 90i Microscope. Images were captured with a NIKON colour camera (24 bit), using aplan Apo NIKON 60X and 100X objective(s). Fluorescence was recorded at 488 nm (green; FITC), and 543 nm (red; rhodamine isothiocyanate B-R18). The images were adjusted and assembled in Adobe Photoshop 10.0. B-R18 and FITC fluorescence levels were quantified using ImageJ on projected confocal stacks, analyzing single confocal slices of identical sizes from within the pericellular region of 10 cells. Quantification of fluorophore colocalization in confocal stacks was done using NIS-Elements AR 3.2 software (Nikon). Pearson's colocalization coefficients (r_p_) were calculated from quantitative data obtained from five confocal stacks.

### Statistical analysis of data

Statistical analysis of the data was performed using GraphPad Prism 4.03 (GraphPad). Data are expressed as means ± standard errors of the means (SEM). Means were compared using using the two-tailed Students t-test. *P* values of less than 0.05 were regarded as statistically significant.

## Results

### Association of α-toxin with *S. aureus* MVs

**Figure 1:**
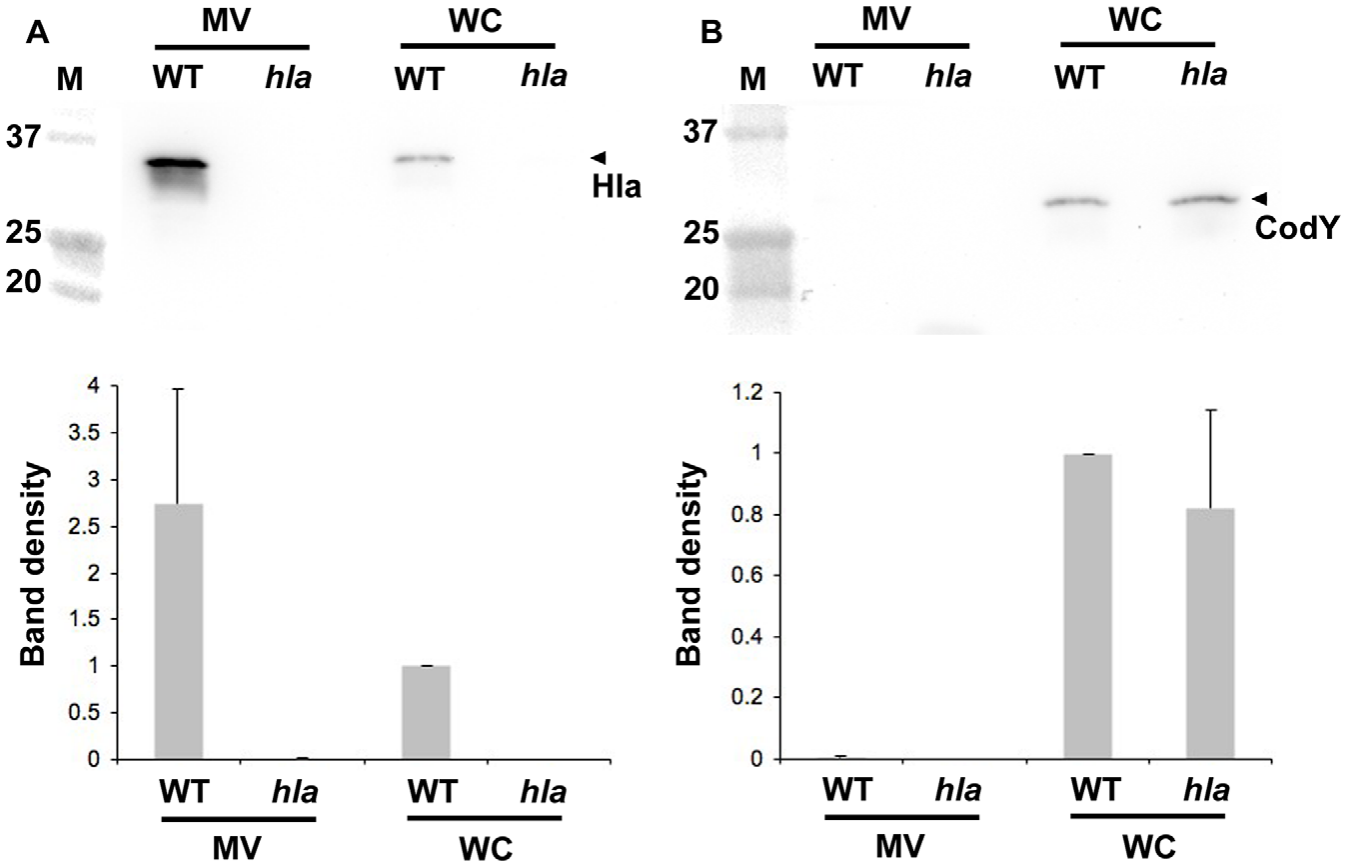
Detection of α-toxin in *S. aureus* MV preparations. Immunoblot detection of α-toxin (Hla; panel A), and CodY (lysis marker; panel B) in MV preparations, and in whole cell (WC) preparation samples from *S. aureus* strain 8325-4 (WT), and from the strain 8325-4 *hla* mutant, DU1090 (*hla*). Polyclonal antisera specific for *S. aureus* α-toxin, and *B. subtilis* CodY, respectively were used for immunoblot detection, and the reactive bands corresponding to these proteins are indicated with an arrowhead. The sizes (kDa) of the proteins in the prestained molecular weight marker (M) are indicated along the left sides. Protein samples equal to 10 µg were applied on the gels. Bar graphs indicate results of densitometric analysis of the immunoblots. Shown are the means ± SEM of relative band density for Hla (A) and CodY (B) from three independent experiments. Data were normalized to the whole cell lysate of the parental strain.

**Figure 2:**
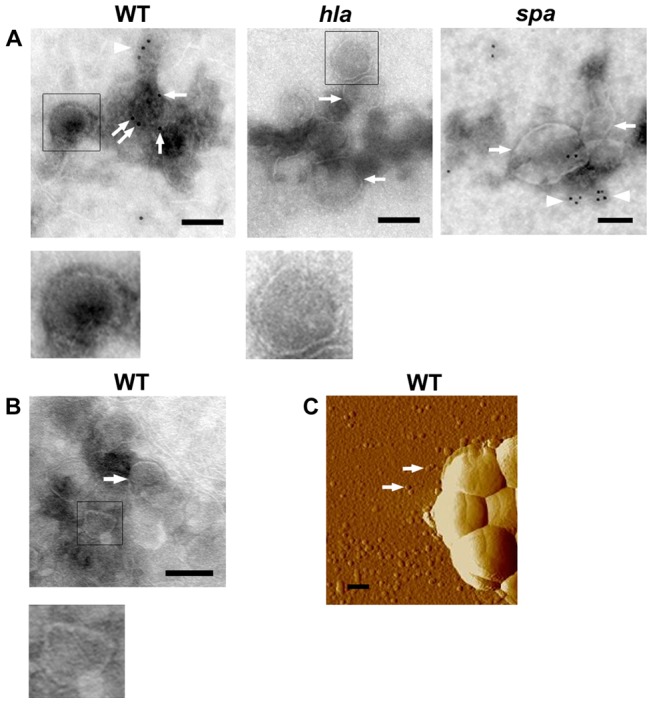
Detection of α-toxin in association with *S. aureus* MVs. Electron microscopy and immunogold-labeling of α-toxin, using a polyclonal antiserum specific for *S. aureus* α-toxin (A), or *B. subtilis* CodY (B). Immunoelectron micrographs of MVs isolated from strain 8325-4 (WT), DU1090 (*hla*), and WA764 (*spa*) are shown. Examples of vesicle structures are indicated by arrows, or highlighted by square boundaries and also shown in larger magnification below the corresponding micrograph. Arrows also indicate gold particles surrounding one 8325-4 (WT) vesicle structure in panel A. Gold particles associated with disrupted vesicle structures are indicated by arrowheads. Bars  = 100 nm. (C) Atomic force micrograph of strain 8325-4 (WT) cultivated on agar. Arrows indicate examples of the released MVs. Bar  = 300 nm.

To investigate if α-toxin is associated with *S. aureus* membrane-derived vesicles, MVs were isolated from strain 8325-4 and its *hla* mutant derivative, DU1090. Western immunoblotting using a polyclonal α-toxin-specific antiserum revealed the presence of α-toxin in both vesicle preparations and whole cell extracts from strain 8325-4, but not in those from the *hla* mutant (Fig. 1A), indicating that at least a fraction of the secreted α-toxin was associated with MVs. On the other hand, the cytoplasmic protein CodY, used as a lysis marker analogously to earlier studies [39], was found only in whole cell lysates of strain 8325-4 and DU1090, and not in MV preparations (Fig. 1B). This ruled out contamination of MV preparations from the cytoplasmic fraction of the bacterial cells. The association of α-toxin with MVs was further supported by electron microscopy analysis using immunogold-labeling and an antiserum specific to α-toxin. As shown in Fig. 2A, gold particles were observed in association with MVs obtained from the wild type strain 8325-4. We also observed that some MVs were ruptured when the samples were mixed with antiserum, and that there was an apparent deposition of gold particles in areas where the vesicle structures seemed disrupted (Fig. 2A). A similar result was obtained with MVs from the *spa* mutant, WA764 (Fig. 2A), which was mainly included to rule out that the IgG-binding activity of protein A contributed to the deposition of gold particles in association with the vesicles. On the other hand, very few gold particles were associated with MVs isolated from the *hla* mutant, DU1090 (Fig. 2A), or observed when strain 8325-4 MVs were assessed using a control antibody, i.e. the antibody specific to the cytoplasmic protein CodY (Fig. 2B). In contrast to the immunogold-EM experiments, AFM revealed that seemingly intact MVs were abundant in samples not subject to antibody treatment (Fig. 2C).

**Figure 3:**
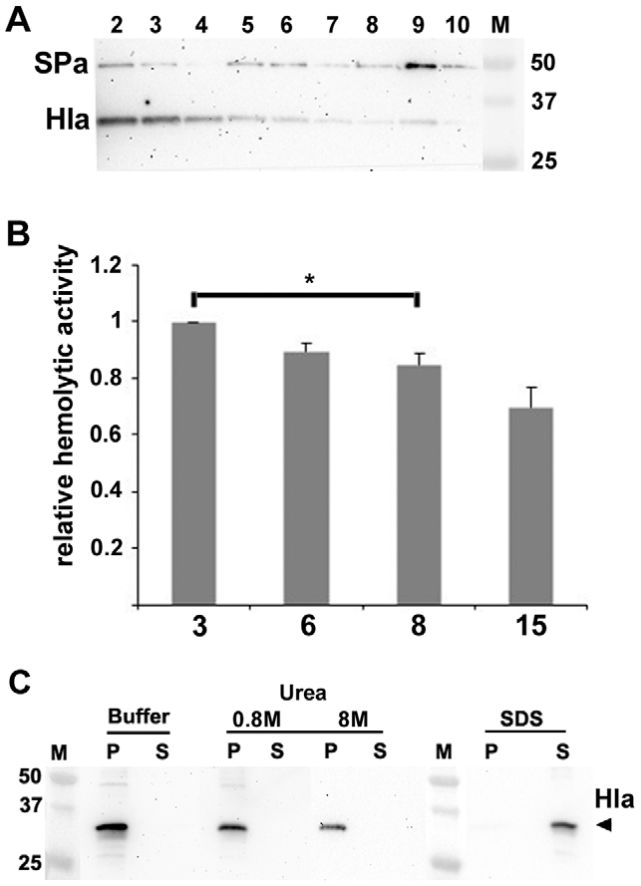
Tight association of α-toxin with *S. aureus* MVs. (A) Immunoblot detection of α-toxin (Hla) and protein A (SPa) in density gradient fractions of MVs from strain 8325-4. Fractions (15 µl applied on the gel) are numbered from left to right (2–10) according to increasing density. A polyclonal antiserum specific for α-toxin was used for immunoblot detection. The sizes (kDa) of the proteins in the prestained molecular weight marker (M) are indicated along the right side. (B) Relative hemolytic activity of density gradient fractions 3, 6, 8, and 15, respectively, as determined using an *in vitro* assay (20% rabbit erythrocytes). Data were normalized to the activity of fraction 3, having the highest α-toxin/protein A-ratio. Shown are the means ± SEM for three independent experiments. *P<0.03, fraction 3 activity vs the activity of either of the other tested fractions. (C) Dissociation assays using MVs isolated from strain 8325-4. An MV preparation in PBS was treated for 60 min on ice in the presence of: PBS (buffer), urea (0.8 M and 8 M), or SDS (1%), respectively. The resulting pellets (P) and supernatants (S) after centrifugation were analyzed by immunoblotting, using a polyclonal anti-α-toxin (Hla) antiserum.

To corroborate our observations, we further assessed the association of α-toxin with *S. aureus* MVs using density gradient fractionation of MV preparations (Materials and Methods). As comparison, we also monitored the presence of protein A in gradient fractions as this protein was earlier found in *S. aureus* MV preparations using mass spectrometry and immunoblotting [8], [23]. According to immunoblotting and densitometric analysis (Fig. 3A), there was an approximately 1∶1 ratio of α-toxin and protein A in gradient fractions 5–7, whereas the relative abundance of α-toxin was higher in the lower-density fractions 2–4 (α-toxin/protein A ratio ∼9∶1 in fraction 3). In accordance with the higher α-toxin/protein A ratio, such fractions exhibited a higher relative hemolytic activity towards rabbit erythrocytes as judged by an *in vitro* assay (Materials and Methods) (Fig. 3B), suggesting that MV-associated α-toxin may be biologically active. In this assay, gradient fraction 15, which exhibited no detectable levels of α-toxin as judged by immunoblotting (data not shown) represented a control. To investigate if α-toxin could be tightly associated with *S. aureus* MVs we used a dissociation assay (Materials and Methods). As indicated in Fig. 3C, after treatment of strain 8325-4 MVs with urea (0.8 or 8 M), α-toxin was mainly recovered with MVs in the pellet. On the other hand, after solubilization of the MVs with SDS (final concentration 1%), α-toxin was completely dissociated, i.e. it could not be detected in the pellet, but was instead released and remained in the supernatant after the subsequent centrifugation (Fig. 3C). These findings suggest that α-toxin was tightly associated with the MVs since the toxin was not extensively dissociated by urea disruption. Hence, taken together our results are consistent with a fraction of α-toxin being associated with membrane-derived vesicles.

### MV-associated α-toxin is biologically active and contributes to cytotoxicity towards HeLa cells

**Figure 4:**
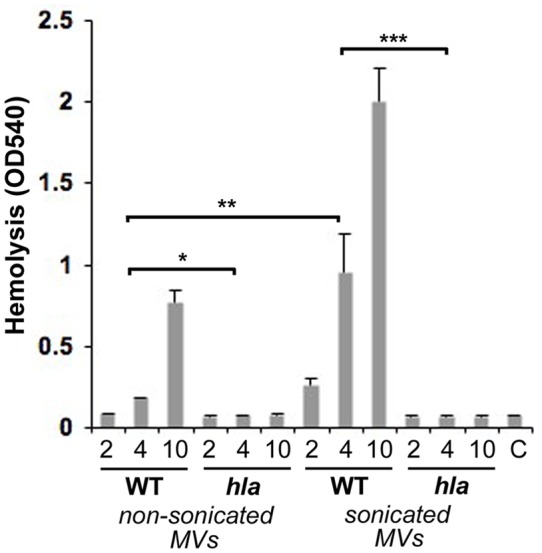
MV-associated α-toxin is biologically active. Hemolytic activity *in vitro* of MVs isolated from *S. aureus* strains 8325-4 (WT), and DU1090 (*hla*), respectively. Rabbit erythrocytes (100% in PBS) were incubated for 60 min with MVs or with MVs disrupted by sonication (2, 4, and 10 µg protein as indicated). Control treatment (C) erythrocytes incubated with PBS. Shown are the means ± SEM for three independent experiments. *P<0.02, 8325-4 MVs vs DU1090 MVs for all tested concentrations; **P<0.05, sonicated vs non-sonicated strain 8325-4 MVs for all tested concentrations; ***P<0.03, 8325-4 MVs vs DU1090 MVs for all tested concentrations.

**Figure 5:**
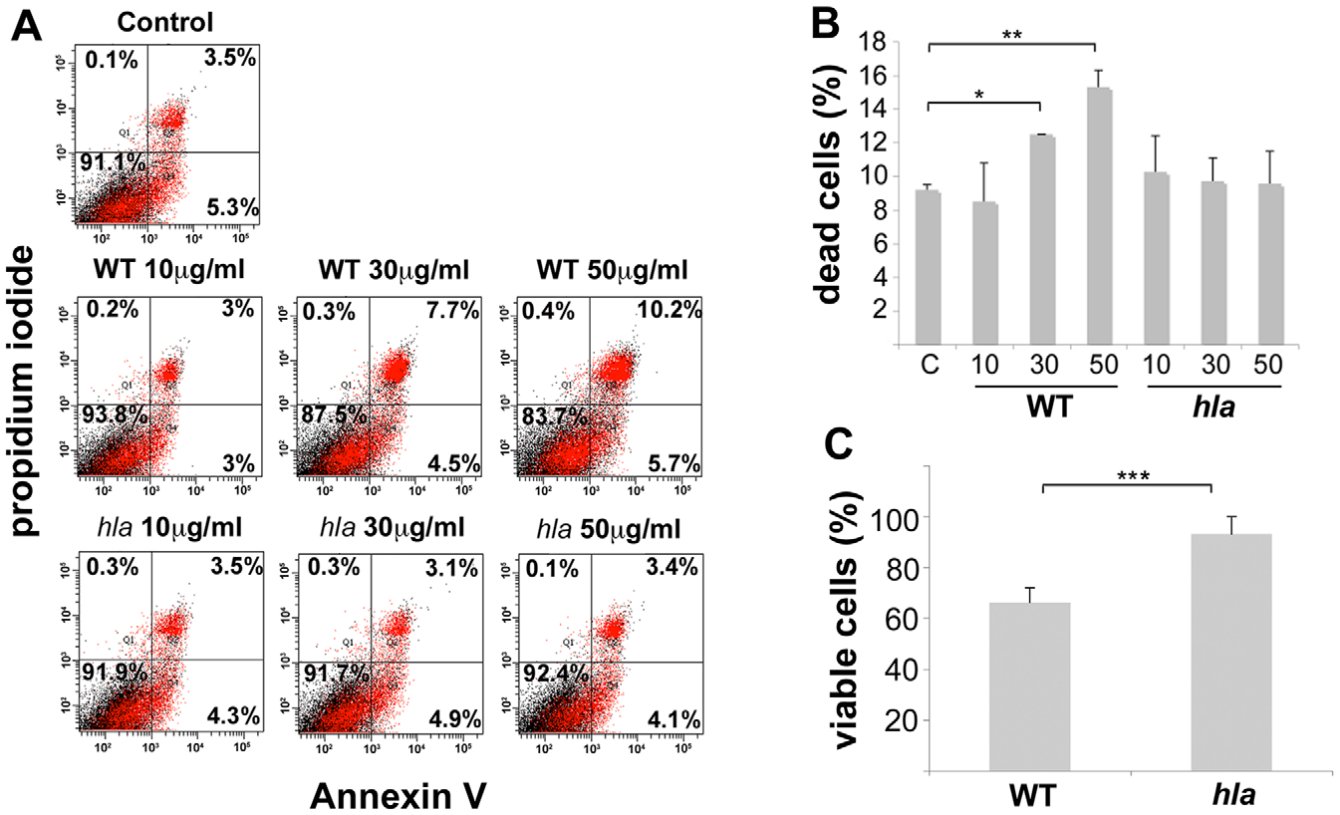
MV-associated α-toxin contributes to cytotoxicity on HeLa cells. HeLa cells were treated with different concentrations of *S. aureus* strain 8325-4 (WT) and DU1090 (*hla*) MVs as indicated for 24 h. Control treatment  =  HeLa cells incubated without vesicles unless specified otherwise. (A) Flow cytometric analysis of cell death. Graphical displays show data from a representative experiment. In each display, the lower right quadrant represents apoptotic cells (Annexin V^+^/PI^−^), and the upper left and right quadrants represent necrotic cells (Annexin V^+^/PI^+^, and Annexin V^−^/PI^+^). (B) Results from two independent flow cytometry experiments. Shown are the means ± SEM of apoptotic + necrotic cells (%) for treatment with 10, 30, and 50 µg/ml MVs. *P = 0.008, 8325-4 MVs vs Control (C); **P = 0.028, 8325-4 MVs vs Control. Panel C: Quantification of neutral red uptake in HeLa cells incubated with MVs isolated from strain 8325-4 (WT), and DU1090 (*hla*). The results shown are means ± SEM from six independent experiments of viable cells (%) for treatment with MVs (50 µg/ml protein). Data were normalized to control treatment (PBS). ***P = 0.013, 8325-4 MVs vs DU1090 MVs.

To confirm that MV-associated α-toxin was biologically active, we assessed the hemolytic activity of strain 8325-4 and DU1090 (*hla*) MVs using rabbit erythrocytes. This provided clear support for an α-toxin-dependent hemolytic activity of MVs. Notably, there was significant dose-dependent hemolysis of rabbit erythrocytes using strain 8325-4 vesicles, whereas MVs from the *hla* mutant, DU1090 exhibited very low activity (Fig. 4). Moreover, the hemolytic activity was considerably enhanced (approximately two- to five-fold) in sonicated relative to non-sonicated vesicle samples from strain 8325-4, whereas this was not seen using DU1090 MVs (Fig. 4). This would be consistent with the liberation of a fraction of α-toxin enclosed in the vesicles. These findings prompted us to assess if MV-associated α-toxin may also contribute to cytotoxic effects on cultured human cells. For this, HeLa cells were treated with different concentrations of strain 8325-4 and DU1090 MVs, respectively for 24 h, and then stained with Annexin V and PI and analyzed by flow cytometry (Fig. 5A, and B). This revealed that strain 8325-4 MVs in contrast to MVs of the *hla* mutant, DU1090, induced a dose-dependent increase (up to 16.3%) in the proportion of dead HeLa cells relative to cells subject to control treatment. To corroborate this observation, the proportion of viable HeLa cells after treatment with MVs for 24 h was determined using neutral red staining (Fig. 5C). This indicated that the fraction of viable HeLa cells was significantly lower after treatment with strain 8325-4 MVs (∼66%) compared to when vesicles from the *hla* mutant, DU1090 were used (∼93%). Hence, we concluded that MV-associated α-toxin was biologically active, contributing to cytotoxic effects causing death of HeLa cells.

### Cholesterol-dependent fusion of *S. aureus* MVs with the host cell plasma membrane

**Figure 6:**
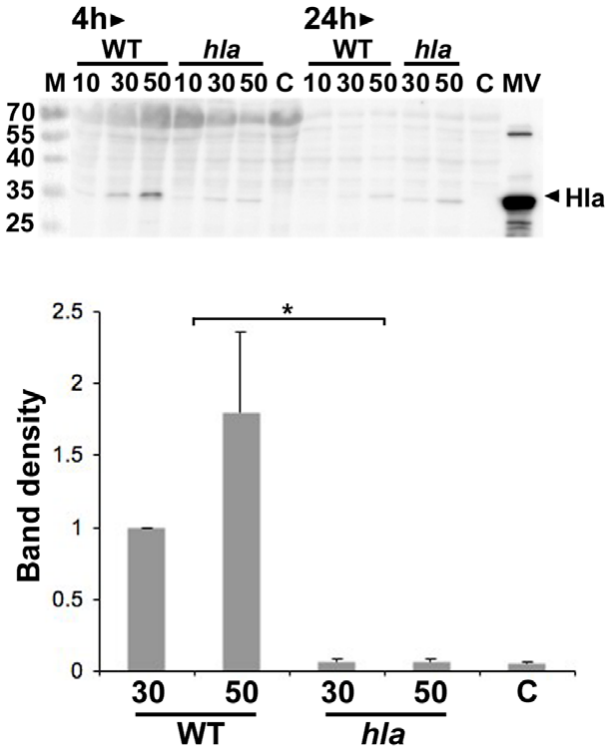
Accumulation of α-toxin in whole cell extracts of HeLa cells treated with *S. aureus* MVs. Immunoblot detection using a polyclonal antiserum specific for *S. aureus* α-toxin. HeLa cells were incubated 4 h and 24 h with MVs obtained from strain 8325-4 (WT), and DU1090 (*hla*) (10, 30, and 50 µg/ml protein as indicated). Whole cell extract samples equivalent to approximately 8,000 lysed HeLa cells were loaded on the gel. Control treatment  =  HeLa cells treated with PBS instead of MVs (C). An MV preparation sample from strain 8325-4 (∼10 µg loaded in the lane denoted MV) served as a positive control. The sizes (kDa) of proteins in the prestained molecular weight marker (M) are specified along the left side. Bar graphs indicate results of densitometric analysis of the immunoreactive band corresponding to α-toxin (also indicated by an arrowhead in the figure). Shown are the means ± SEM of relative band density from three independent experiments for treatment of HeLa cells with strain 8325-4 (WT), and DU1090 (*hla*) MVs (30 and 50 µg/ml protein as indicated) for 24h. Data were normalized to treatment with 8325-4 MVs at a concentration of 30 µg/ml protein. *P<0.04, 8325-4 MVs vs DU1090 MVs for both tested concentrations. The identity of the protein having a slightly smaller molecular size than α-toxin is not known.

**Figure 7:**
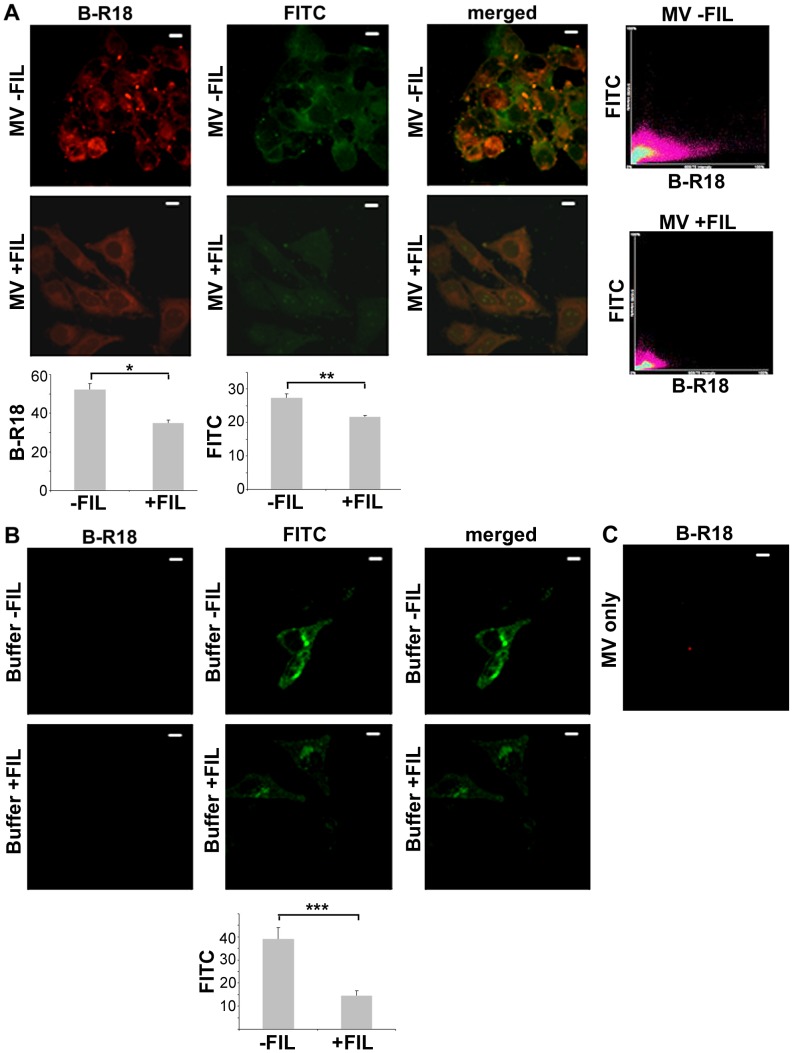
Cholesterol-dependent fusion of *S. aureus* MVs with HeLa cells. Localization of rhodamine B-R18-labeled MVs (red fluorescence used as a readout for MV fusion with the host cell plasma membrane), and FITC-conjugated lipid raft marker CtxB (green fluorescence) in HeLa cells after 30 min of incubation with membrane-derived vesicles obtained from strain 8325-4 (MV; panel A), and with PBS (buffer; panel B). Treatment was done in the absence (−FIL) and in the presence (+FIL), respectively, of the cholesterol-sequestering agent Filipin III (final concentration 10 µg/ml). Bar graphs show quantitative analysis of red (B-R18) and green (FITC) fluorescence in treated HeLa cell samples. Values represent arbitrary units of pixel intensity for red and green fluorescence determined using ImageJ, and shown are the means ± SEM of data collected from 10 cells. *P = 0.0001, **P<0.0001, and ***P = 0.0002, for treatment in the absence vs presence of Filipin III. The merged images show the labeling with both fluorescent dyes. The scattergrams in panel A with red (B-R18) and green (FITC) pixels plotted on graphs were used to obtain the colocalization coefficient (r_p_) between MVs and CtxB in the HeLa cells treated with strain 8325-4 MVs for 30 min. (C) B-R18-labeled MVs alone. Magnification: 1000×. Bars  = 10 µm.

Consistent with the α-toxin-dependent induction of cytotoxic effects on HeLa cells by MVs (Fig. 5), there was a dose-dependent increase in the abundance of α-toxin in whole cell extracts of HeLa cells incubated with different amounts of strain 8325-4 MVs (Fig. 6). In contrast, this was not seen when cells were subject to control treatment (PBS, or vesicles from the *hla* mutant DU1090) (Fig. 6). This suggests that the MVs may play a role in delivering α-toxin to host cells. To investigate how *S. aureus* MVs may interact with host cells, MVs were labeled with rhodamine isothiocyanate B-R18, which is dequenched upon fusion with the host cell plasma membrane, resulting in red fluorescence [35], [36]. According to confocal microscopy studies there was a clear increase in red fluorescence in HeLa cell samples incubated with B-R18-labeled vesicles for 30 min (Fig. 7A), whereas in contrast the red fluorescence remained at background levels in HeLa cell samples treated with buffer instead of MVs (Fig. 7B), and in samples containing only B-R18-labeled MVs (Fig. 7C). This is consistent with the notion that the *S. aureus* MVs fused with the HeLa cell plasma membrane. To assess if this interaction of MVs with the HeLa cells was dependent on cholesterol-rich domains in the plasma membrane, HeLa cells were treated with B-R18-labeled MVs for 30 min in the presence and absence, respectively, of the cholesterol-sequestering agent Filipin III. FITC-conjugated CtxB subunit (green fluorescence) was included in the assay as a plasma membrane lipid raft marker [37]. As evidenced by a clear reduction in red fluorescence (Fig. 7A), Filipin III eliminated the fusion of MVs with HeLa cells. Moreover, Filipin III inhibited the binding of CtxB to lipid raft microdomains as indicated by reduced levels of green fluorescence in HeLa cell samples treated with either MVs (Fig. 7A) or buffer (Fig. 7B). However, as determined by confocal microscopy, there was no apparent colocalization of CtxB with B-R18-labeled MVs in HeLa cells within 30 min of incubation (r_p_<0.5) (Fig. 7A). Hence, these results together are consistent with fusion of *S. aureus* MVs with the host cell plasma membrane in a cholesterol-dependent manner, albeit not specifically localizing to lipid raft microdomains. In accordance with the induction of cell death by MVs (Fig. 5), HeLa cells incubated with MVs for 30 min showed extensive rounding and cell shrinkage (Fig. 7A). Interestingly, this was not seen when HeLa cells were treated with vesicles in the presence of Filipin III (Fig. 7A), suggesting the possibility that fusion of MVs with the plasma membrane may be important for host cell cytotoxicity.

## Discussion

In this study we have identified *S. aureus* membrane-derived vesicles as a vehicle to deliver biologically active α-toxin to human cells. Similar to several previous studies on vesicle-associated proteins [2], [6], [28], [29], [40], [41], we used immunoelectron microscopy, a density gradient, and a dissociation assay to separate the vesicles from loosely associated proteins, and to demonstrate the tight association of α-toxin with *S. aureus* MVs. Our immunogold-EM data, revealing an apparent deposition of gold particles in areas where the vesicle structures seemed disrupted, are in concordance with earlier studies on *Campylobacter jejuni* cytolethal distending toxin (CDT), *Escherichi coli* α-hemolysin (HlyA), and hemolysin of enterohemorrhagic *E. coli* (EHEC) isolates (EHEC-Hly) [2], [6], [42]. Our observation of disrupted MVs in the presence of antiserum is consistent with serum protein-mediated destabilization of *Cryptococcus neoformans* and *Bacillus anthracis* extracellular vesicles [43]. In two of the above studies [2], [6] it was proposed that at least a fraction of the toxin was enclosed in vesicles and hence not accessible to the antiserum used in immunogold-EM. This would agree with our present finding that sonicated MVs exhibited enhanced hemolytic activity relative to non-sonicated vesicles.

Differences in size and density (i.e. lipid:protein ratio) observed for vesicles reflect differences in vesicle composition [44]. The higher abundance of α-toxin relative to protein A in the low density fractions (high lipid:protein ratio [45]) after density gradient fractionation of vesicles might be explained by the intrinsic features of α-toxin, i.e. membrane lipids seem to be central to the interaction of the toxin with the eukaryotic cell [22], [46]. Hence, it cannot be excluded that there are MVs enriched with α-toxin, which similar to vesicles carrying *E. coli* HlyA [2], may be distinct from MVs containing no or low levels of the toxin, showing a lower density. This remains to be tested.

Our present results, revealing MV-associated α-toxin, are in accordance with the identification of this toxin in vesicle preparations from *S. aureus* strains ATCC14458 and 06ST1048 using mass spectrometry [8], [23]. We used rabbit erythrocytes, and HeLa cells in our experiments demonstrating that MV-associated α-toxin was biologically active. Albeit only a minor fraction of secreted α-toxin appear to be vesicle-associated (B. Thay, S.N. Wai, and J. Oscarsson, unpublished results), similar to HlyA of extraintestinal pathogenic *E. coli* isolates [2], our present results strongly supported that α-toxin is the main *S. aureus* MV-associated protein that is responsible for erythrocyte lysis. The induction of cell death of HeLa cells by strain 8325-4 MVs is in accordance with earlier observations incubating strain ATCC14458 MVs with HEp-2 cells [23]. Our present findings with MV-associated α-toxin is as far as we know the first demonstration of an *S. aureus* effector protein contributing to the induction of host cell death by MVs. This activity of MV-associated α-toxin is consistent with earlier evidence from studies treating Jurkat T cells with wild type and *hla* mutant *S. aureus* strains, revealing that α-toxin is the major *S. aureus* mediator of caspase activation and apoptosis [18].

Our current data revealing the accumulation of α-toxin in whole cell extracts of MV-intoxicated HeLa cells are consistent with the earlier demonstrated delivery of protein A to HEp-2 cells [23]. Hence, it is conceivable that *S. aureus* MVs may simultaneously carry multiple virulence factors to host cells. In this work we have also suggested some of the mechanisms involved in the delivery of α-toxin via MVs to the target cells. According to our findings monitoring the dequenching of a fluorescent probe, the MVs interacted with HeLa cells via a mechanism of MV fusion with the plasma membrane, which was detected after 30 min of incubation. Apparently, plasma membrane cholesterol was required for this interaction as it could be abolished using the cholesterol-sequestering agent Filipin III. As this drug also seemed to prevent MV-associated cytotoxic effects on the HeLa cells, fusion of *S. aureus* MVs with the target cell plasma membrane may serve as an important mechanism to induce host cell death. It cannot therefore be excluded that Filipin III may have blocked the interaction of MVs with a putative host cell receptor, although this remains to be tested. Moreover, the cholesterol-depletion may have resulted in reduced α-toxin activity, consistent with earlier observations [46]. Our results assessing the colocalization of labeled vesicles with a lipid raft marker (CtxB) suggest that *S. aureus* MVs did not require cholesterol-rich lipid raft microdomains *per se* to interact with the host cells. A dependence on lipid rafts was proposed earlier based on the observation that cell death of Hep-2 cells treated with *S. aureus* MVs, and delivery of protein A, was abolished in the presence of the cholesterol-binding compound methyl-β-cyclodextrin [23], albeit in that study it was not elucidated if the MVs colocalized with lipid raft microdomains of the Hep-2 cells. To our knowledge, *S. aureus* MVs are the first example of MVs from a Gram-positive organism that can deliver virulence factors to host cells via membrane fusion, whereas this mode of delivery of effector proteins has been recognized for OMVs from some Gram-negative bacteria, e.g. *Aggregatibacter actinomycetemcomitans*, and *Pseudomonas aeruginosa* [29], [35].

The importance of α-toxin as a virulence factor has been supported using a number of infection models, including experimental brain abscess, keratitis, and pneumonia [47]–[49]. Hence, MV-associated delivery of α-toxin might be advantageous for *S. aureus* in several different types of infections. On the other hand, studies have been published either supporting or arguing against the notion that presence of α-toxin is incompatible with an intracellular persistence of *S. aureus*, although study outcomes seem to partly depend on the target cell type used [50]–[52]. Further to causing possible detrimental effects during intracellular persistence, it cannot be excluded that MV-mediated delivery of α-toxin may cause perturbations of cellular functions in more subtle ways. A mechanism for such modulation was earlier demonstrated with *E. coli* OMVs, which after fusion with the cell membrane, and/or internalization of OMVs, delivered sublytic concentrations of the pore-forming cytolysin, ClyA, resulting in altered Ca^2+^ homeostasis in primary renal epithelial cells [53]. Such modulation by MV-associated α-toxin could be of particular importance in chronic *S. aureus* infections, which seem to rely on the ability of *S. aureus* to persist in human host cells [54], [55].

In conclusion, our results provide a molecular basis for the MV-mediated delivery of biologically active α-toxin to human host cells. As MVs are evidently produced *in vivo* [23], it will be of interest to assess the role of the MV-associated delivery of α-toxin in different models of infection.
